# Anomalies of the Portal Venous System in Dogs and Cats as Seen on Multidetector-Row Computed Tomography: An Overview and Systematization Proposal

**DOI:** 10.3390/vetsci6010010

**Published:** 2019-01-22

**Authors:** Giovanna Bertolini

**Affiliations:** San Marco Veterinary Clinic and Laboratory, via dell’Industria 3, 35030 Veggiano, Padova, Italy; bertolini@sanmarcovet.it; Tel.: +39-049-856-1098

**Keywords:** portal system, portal vein, portosystemic shunt, portal hypertension, computed tomography

## Abstract

This article offers an overview of congenital and acquired vascular anomalies involving the portal venous system in dogs and cats, as determined by multidetector-row computed tomography angiography. Congenital absence of the portal vein, portal vein hypoplasia, portal vein thrombosis and portal collaterals are described. Portal collaterals are further discussed as high- and low-flow connections and categorized in hepatic arterioportal malformation, arteriovenous fistula, end-to-side and side-to-side congenital portosystemic shunts, acquired portosystemic shunts, cavoportal and porto-portal collaterals. Knowledge of different portal system anomalies helps understand the underlying physiopathological mechanism and is essential for surgical and interventional approaches.

## 1. Introduction

**Figure 1:**
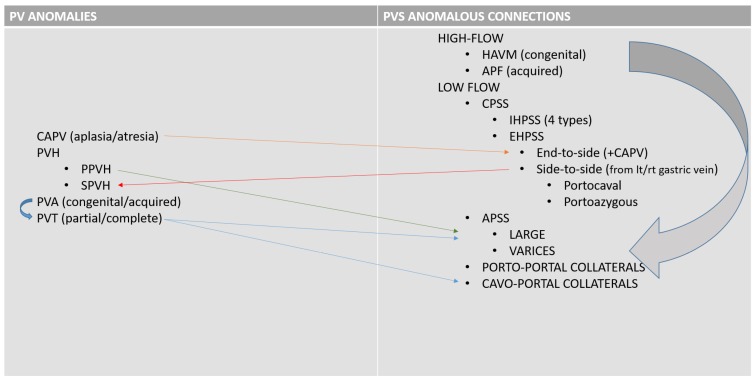
Schematization of the anomalies of the portal vein (left) and anomalous connections involving the portal venous system. Arrows indicate possible relationships between various congenital and acquired anomalies. CAPV, congenital absence of portal vein; PVH, portal vein hypoplasia; PPVh, primary portal vein hypoplasia; SPVH, secondary portal vein hypoplasia; PVA, portal vein aneurysm; PVT, portal vein thrombosis; HAVM, hepatic arteriovenous malformation; APF, arterioportal fistula; CPSS; congenital portosystemic shunt; IHPSS, intrahepatic portosystemic shunt; EHPSS, extrahepatic portosystemic shunt; APSS, acquired portosystemic shunt.

The portal venous system is essential for the maintenance of the liver mass and function in mammals. The portal system collects blood from major abdominal organs (i.e., gastrointestinal tract, pancreas, spleen) delivering nutrients, bacteria and toxins from the intestine to the liver. In addition, the portal blood carries approximately from one-half to two-thirds of the oxygen supply to the liver and specific hepatotrophic factors [1,2]. The portal blood is detoxified by the hepatocytes and then delivered into the systemic circulation via the hepatic veins and caudal vena cava [3]. Gross anatomic connections between the portal system and systemic venous circulation, at any level or other portal system anomalies can have serious clinical consequences [4,5,6,7,8]. Anomalies of the portal vein and portal system in small animals have been increasingly detected in recent years with the growing availability of advanced imaging techniques in veterinary practice [9,10,11,12,13]. A broad spectrum of congenital and acquired disorders may affect the portal venous system in dogs and cats. Congenital disorders of the portal system reflect a variety of embryonic or foetal disturbances, which may be isolated or combined in complex vascular patterns and may be cause of acquired vascular disorders. For example, congenital arterioportal communications are associated with acquired portal collaterals that form in response to the portal hypertension [10,14] (Figure 1). Knowledge of the normal anatomy and congenital and acquired patterns and their consequences is of great importance for clinical decision-making for liver surgery and for planning interventional procedures such as transvenous portosystemic shunts or transarterial arterioportal fistulas coil embolization [15,16]. First line imaging evaluation of the portal venous system in small animals is usually performed with colour Doppler ultrasonography. Invasive techniques (e.g., splenoportography) used to obtain more detailed morphological information, are nowadays replaced by computed tomography (CT) or magnetic resonance imaging (MRI) angiography [17,18,19,20,21].

Multidetector-row computed tomography (MDCT) angiography is now widely considered to be the method of choice for the diagnosis and monitoring of portal vascular anomalies in veterinary practice [22,23,24] MDCT provides excellent visualization of complex vascular anomalies and a comprehensive overview of the entire portal system and allows simultaneous evaluation of other vascular and non-vascular structures of the abdomen.

In this article, we present an overview of normal anatomy of the portal system, as seen by MDCT angiography and propose a systematization of congenital and acquired pathological conditions in dogs and cats. 

All collection procedures presented here were performed solely for the dog’s benefit and for standard diagnostic and monitoring purposes. Previous informed written consent was obtained from all dog owners. All the procedures performed complied with the European legislation “on the protection of animals used for scientific purposes” (Directive 2010/63/EU) and with the ethical requirement of the Italian law (Decreto Legislativo 04/03/2014, n. 26).

## 2. Brief Anatomy Background

**Figure 2:**
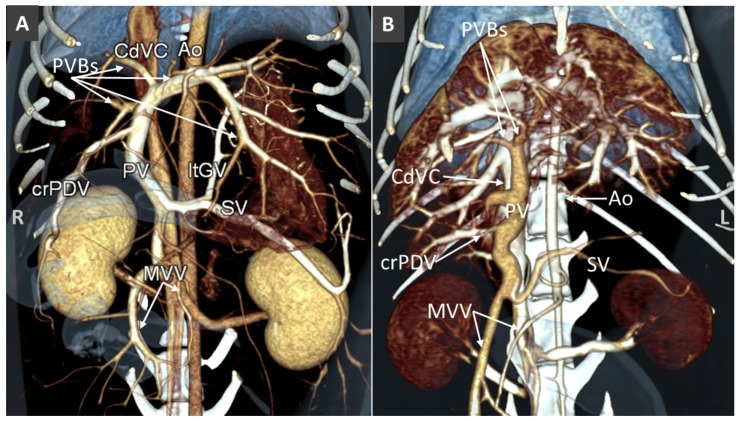
Volume rendered images (ventral views) of normal portal venous system in a dog (**A**) and a cat (**B**). Ao, aorta; CdVC, caudal vena cava; PVBs, portal vein branches; PV, portal vein; ltGV, left gastric vein; crPDV cranial pancreatic duodenal vein; SV, splenic vein; MVV, mesenteric veins.

The portal system forms by the confluence of cranial and caudal mesenteric veins, which drain most of the intestinal tract. From the left side the splenic vein, which accompanies the corresponding artery and receives the left gastric vein, contributes to the portal vein formation. From the right side, the portal vein receives the gastroduodenal vein, right gastric and gastroepiploic veins before entering the liver parenchyma. At the hepatic porta, the portal vein divides into right and left branches (Figure 2) [25]. At this level, the portal vein pattern is almost constant, while the number of the portal branches entering individual liver lobes can vary among subjects [26,27]. In general, the right branch is a short venous trunk, which supplies the right portion of the liver. One or two dorsolateral primary branches supply for the caudate process of the caudate lobe and one or three ventrolateral branches ran towards the right lateral lobe of the liver. The left branch of the portal vein provides several primary branches supplying the central and left portions of the liver, comprising the right medial, quadrate, left medial and left lateral lobes. In most circumstances, the left portal branch supplies also the dorsal, right lateral liver lobe. One to three small branches for the papillary process of the caudate lobe arise directly from the left branch. The feline portal vein divides into left, central and right portal branches providing intrahepatic subdivisions to the liver lobes. [28,29]. At all sizes and subdivisions, branches of the hepatic artery, portal vein and bile ducts pass in close proximity to one another. Interlobular branches of the hepatic artery and portal vein form hepatic sinusoids, which collect detoxified blood into central veins and finally into the vena cava system.

## 3. Portal System Venous Anomalies (PVSA)

### 3.1. Disorder of the Portal Vein System

#### Congenital Absence of the Portal Vein (Aplasia and Atresia)

Congenital absence of portal vein (CAPV) is a rare condition in which the portal blood bypasses the liver and a splenomesenteric shunt to the systemic circulation is present [30,31]. The CAPV is attributed to excessive involution of the periduodenal vitelline veins or failure of the vitelline veins to establish anastomosis with hepatic sinusoids, which leads to partial (of the extrahepatic portal vein) or complete (involving also the intrahepatic portal component) absence of the portal system (aplasia or agenesis). As a result, the umbilical flow and enterohepatic circulation are disturbed and the portal as well as placental venous flow is shunted systemically. There is close relationship between the vitelline veins and the complex development of the systemic veins likely explaining the occurrence of extrahepatic portosystemic shunt in case of CAPVS [32,33]. In fact, cases of CAPVS in dogs are always described in association to the portal insertion into the caudal vena cava and total diversion of portal blood into the systemic circulation (end-to-side portosystemic shunt) [34,35].

Imaging alone cannot distinguish between complete or incomplete forms of portal vein aplasia. The definitive diagnosis of complete agenesis of the portal system requires additional histological analysis of the hepatic parenchyma that demonstrates the absence of hepatic portal venules within the portal triad [36].

**Figure 3:**
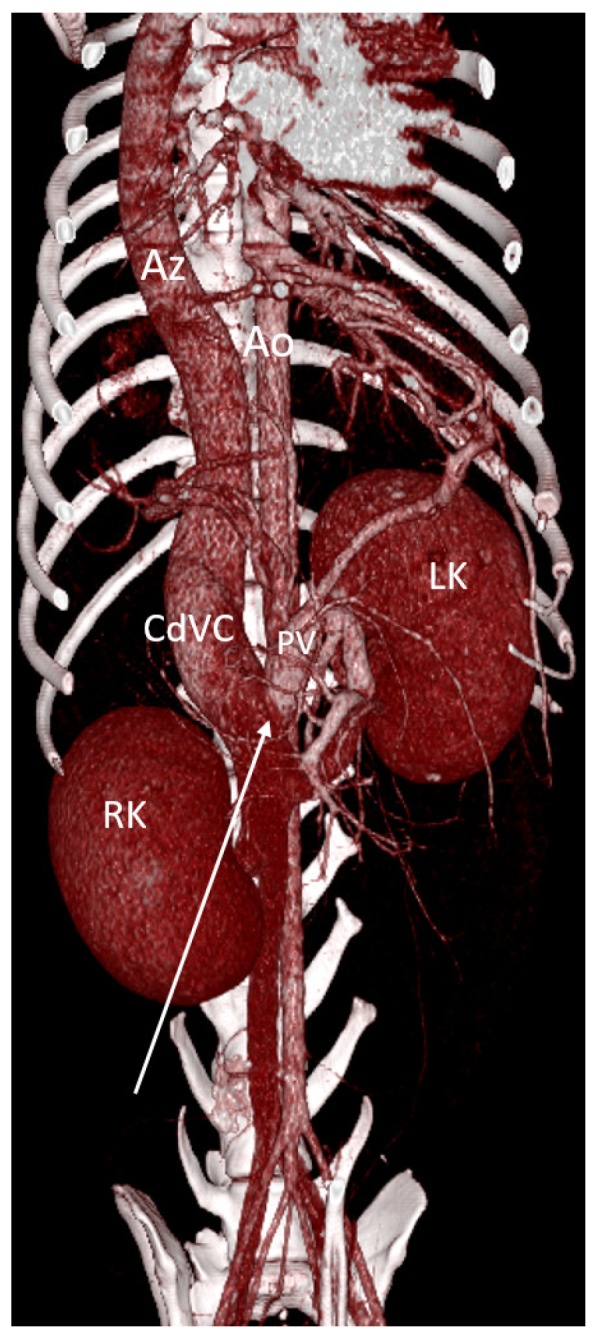
Volume rendered image (ventral view) of a dog with extrahepatic CAPV, portal insertion in the renal segment of the CdVC (arrow). This is an example of end-to-side portosystemic shunt. There is simultaneous azygous continuation of the CdVC due to congenital absence of renal and prehepatic CdVC segments. Moreover, the dog showed situs inversus abdominalis (note the left-sided mesenteric and portal veins, the caudal right kidney and cranial left one). LK, left kidney; RK, right kidney.

In dogs, CAPV has been described in association to other developmental anomalies including situs inversus, congenital heart diseases, vena cava anomalies and polysplenia (Figure 3) [32,37].

**Figure 4:**
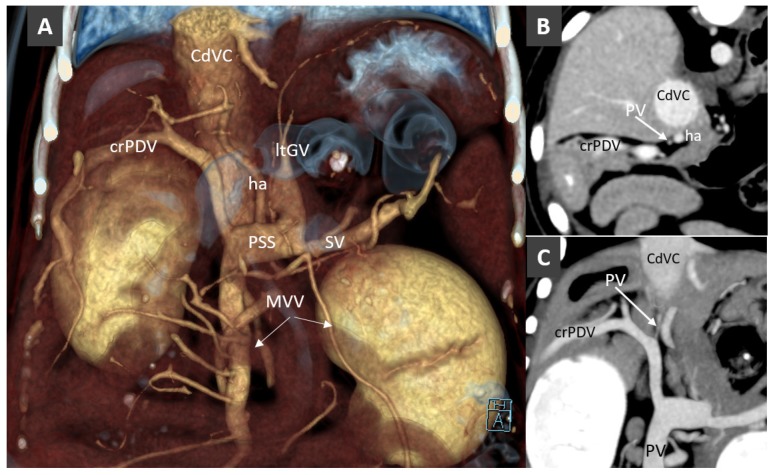
(**A**). Volume rendered image (ventral view) in a Mongrel dog with extrahepatic portosystemic shunt (PSS). The portal vein is not visible. MVV, mesenteric veins; PSS, portosystemic shunt; SV, splenic vein; ha, hepatic artery; ltGV, left gastric vein; crPDV, cranial pancreaticoduodenal vein. (**B**). Transverse view at the porta hepatis of the same dog. An hypoplastic PV is barely visible. (**C**). Dorsal thin-MIP (Maximum Intensity Projection) image in the same dog, showing the thin, hypoperfuse portal vein (portal atresia).

Reduced portal blood flow in the portal vein due to flow diversion through a several types of congenital extrahepatic portosystemic shunt (CPSS) may simulate the absence of the portal vein. An adequate CT portal venous vascular phase may reveal a hypoplastic, hypoperfused portal vein (portal vein atresia) and hepatic portal branches, which makes the patient a candidate for surgical intervention of the portosystemic shunt (Figure 4). On contrary, in case of portal vein aplasia, surgical attenuation of the portosystemic connection will result in fatal acute portal hypertension [6]. Histopathology data may be added to CT data for confirmation of presence or absence of the portal venules within the portal triad. However, smaller or absent portal tract within the liver can be detected in liver biopsy samples of both aplasia and hypoplasia portal vein conditions [38].

### 3.2. Portal Vein Hypoplasia

Portal vein hypoplasia (PVH) refers to a disorder in which microscopic portal veins within the liver are underdeveloped. PVH can be morphological (primary portal vein hypoplasia [PPVH]) or functional (secondary PVH or SPVH) due to a reduction of portal blood flow toward the liver [14,36]. These two conditions (morphological or functional) are not distinguishable either by radiology or histopathology alone. Integration of imaging and histological information is thus mandatory to the diagnosis of PVH and distinction of morphological or functional conditions. In fact, macroscopic disorders causing chronic hypoperfusion of the liver, such as portosystemic shunting, arterioportal fistula (APF) and portal vein thrombosis (PVT), can lead to the same hepatic histological features as PPVH and should be ruled out with imaging [39,40,41].

### 3.3. Portal Vein Aneurysm

**Figure 5:**
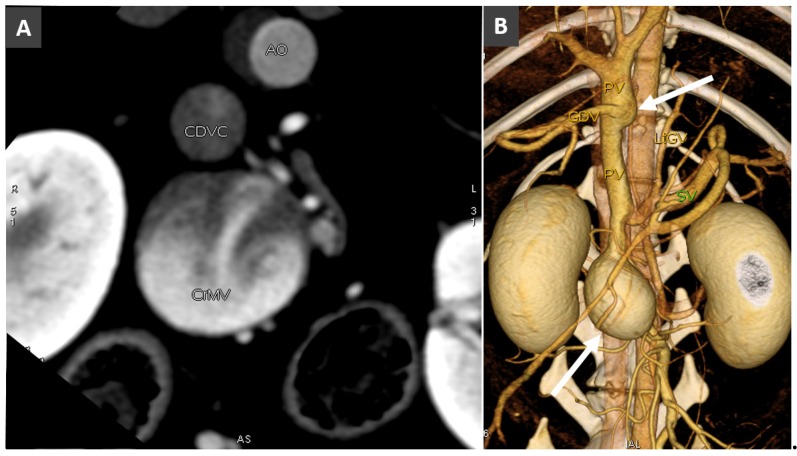
(**A**). Transverse view in a dog with large saccular aneurysm of the caudal mesenteric vein (CrMV) at the confluence with the splenic vein (not visible here). CDVC, caudal vena cava; Ao, aorta. (**B**). Volume rendered image (ventral view) of the same dog, showing the large saccular aneurysm of the cranial mesenteric vein and a fusiform aneurysmal dilatation of the prehepatic portal vein (arrows).

Portal vein aneurysm (PVA) has a reported prevalence of 0.49% in dogs [42]. PVA can be congenital or acquired. Proposed mechanisms for PVA are an inherent weakness of the vessel wall or incomplete regression of the distal part of the vitelline vein. Larger, male dogs are more frequently affected and the boxer breed seems to be predisposed to PVA development. On MDCT images, PVA appears as saccular or fusiform dilatation of the portal vein or its branches within the liver. Extrahepatic PVAs are generally located at the insertion of the gastroduodenal vein into the portal vein. Intrahepatic PVAs occur at bifurcations (Figure 5). Clinical features of PVAs are related to their size and possible complications, such as thrombosis, rupture of the aneurysm and portal hypertension. Acquired portal collaterals may coexist with PVA [42,43] Aneurysm of the mesenteric veins can occur in dogs, alone or in combination with PVA. Pseudoaneurysmal dilatation of portal branches is also detected in cases of intrahepatic CPSS and hepatic arterioportal malformation [44,45].

### 3.4. Portal Vein Thrombosis

Portal vein thrombosis (PVT) refers to the partial or total luminal obstruction of the portal vein, with or without extension to its portal branches within the liver, the splenic vein or the mesenteric vein [46].

**Figure 6:**
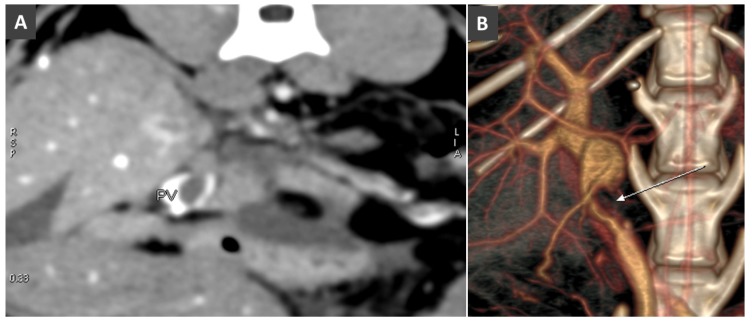
(**A**). Transverse image of the liver at the porta hepatis in a cat with pancreatitis and PV (portal vein) thrombosis (arrow). (**B**). Volume rendered image (ventral view) in the same cat. Note the large filling defect due to the thrombosis (arrow) in the prehepatic PV.

**Figure 7:**
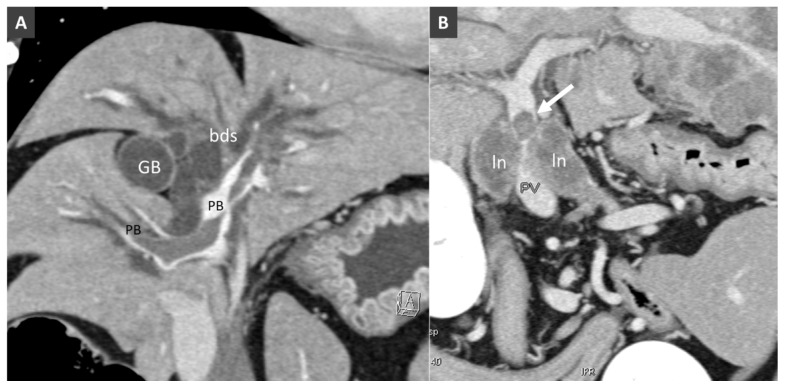
(**A**). Dorsal MPR (multiplanar reformation) view of the liver in a dog with suppurative hepatitis (ln, lymph node). (**B**). Dorsal MPR view in another dog. Note the large filling defect in the portal branches (PB) within the liver. Biliary ducts (bds) are enlarged gallbladder (GB).

Local causes of PVT, such as PVA and vascular invasion from hepatic or pancreatic neoplasia, are easily identified by MDCT. (Figure 6 and Figure 7) [43].

PVT can lead to partial or complete obstruction of the portal lumen. Both, partial and complete PVT, may show peripheral enhancement in post-contrast series [23,42,47]. Partial and complete obstruction should be distinguished, because they can have different clinical consequences. In cases of partial occlusion, some contrast medium passes around the thrombus. In cases of complete obstruction, enhancement of the peripheral rim of the thrombus is likely due to dilatation of the vasa vasorum attempting to recanalize the vessel [23].

Indirect signs of PVT in chronic obstruction are porto-portal collaterals (see below), arterioportal shunts and APSS [10,47] Because of portal vein obstruction, compensatory mechanisms are immediately activated to re-establish the portal flow to the liver and small arterial–portal connections may form within the liver. A second mechanism is the formation of portal venous collaterals. In cases of complete extrahepatic portal vein obstruction, characterized by high pressure in the splanchnic circulation and normal pressure in the hepatic sinusoids, multiple collateral hepatopetal vessels form around the thrombotic segment (see below). 

### 3.5. Anomalous Vascular Connections of the Portal Vein System

The anomalous vascular connections of the portal system can be classified broadly as high-flow and low-flow portal connections. 

#### High-Flow Anomalous Portal Connections 

**Figure 8:**
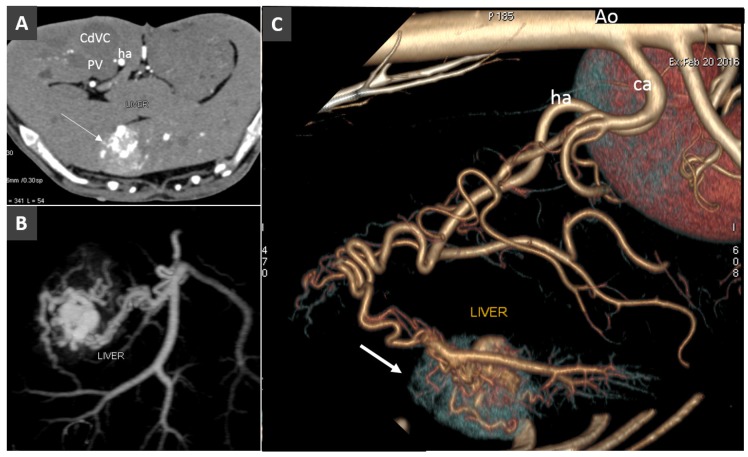
Acquired arterioportal fistula in a dog. (**A**). Transverse view of the liver obtained during early arterial phase (ha, hepatic artery). Note the large hypervascular hepatic lesions (arrow). **B.** Dorsal MIP showing the arterioportal communication. (**C**). Left lateral view of volume rendered image of the same volume (ca, celiac artery). The arrow indicates the focal early enhancement of the liver parenchyma due to the arterioportal communication.

**Figure 9:**
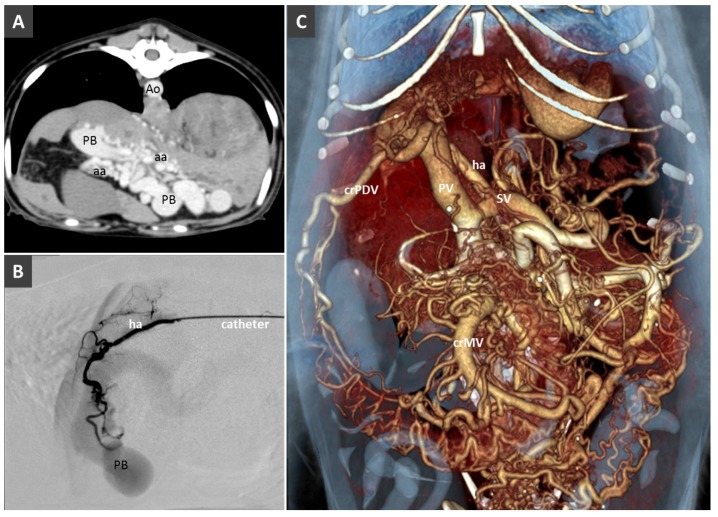
(**A**). HAVM in a young dog with portal hypertension and multiple acquired portal collaterals. Ao, aorta; PB, portal branches; aa, branches of the hepatic artery. **B.** Angiogram before selective transarterial coil embolization through hepatic artery (ha) in the same patient. (**C**). Volume rendered image showing the portal venous system dilatation and multiple portal collaterals. PV, portal vein; ha, hepatic artery; SV, splenic vein; crPDV, cranial pancreaticoduodenal vein.

High-flow portal connections are based upon the presence of an arterial component. These conditions have been rarely described in dogs and cats and different terminology has been used interchangeably causing confusion. High-flow anomalous connections involving the portal venous system include congenital hepatic arteriovenous malformations (HAVMs) and congenital or acquired arterioportal fistulas (APFs) (Figure 8 and Figure 9). HAVMs refers to a congenital disorder in which a plethora of dysplastic arteries drain or shunt directly into arterialized portal vein branches, creating a vascular nidus (arterialization of the portal system) [44,48,49]. Sites of communications between the arterial and portal vessels are often difficult or impossible to determine with non-selective CT angiography [50,51]. Recently, HAVMs have been classified as right divisional or left divisional and the left divisional were subclassified as left medial and left lateral, depending on the site of the efferent portal vein drainage [50]. Although congenital, these anomalies may be clinically silent until hepatoencephalopathy and portal hypertension develop. Imaging of these patients shows direct and indirect signs (ascites and acquired portal collateral circulation). HAVMs can be treated by transcatheter embolization alone or in combination with surgery but they may be difficult to treat definitively [51].

A single APF is a congenital or acquired, communication between a high-pressure hepatic arterial branch and a low-pressure portal branch outside or inside the liver [52]. Penetrating abdominal trauma, including liver biopsy and neoplasia are possible causes of APFs that are generally single and thus easier to treat. Signs of pre-sinusoidal portal hypertension may ensue, such as ascites and acquired portosystemic shunts [14]. Finally, in several hepatic pathological conditions, small or microscopic acquired hepatic peripheral APFs can be identified or suspected in multiphase, thin section MDCT examination of the liver. Their presence can cause areas of early parenchymal opacification (hepatic perfusion disorders) enhancing in late arterial phase, rather than in portal venous phase [22,23] These APFs do not require treatment, as they usually resolve spontaneously or diffuse with the progression of the underlying pathology [53].

### 3.6. Low-Flow Anomalous Portal Connections 

Low-flow anomalous portal connections are congenital and acquired portosystemic shunts, porto-portal collaterals that develop in case of portal vein obstruction and cavo-portal collaterals that for chronic increased resistance at any level of the caudal vena cava. 

#### Congenital Portosystemic Shunt 

**Figure 10:**
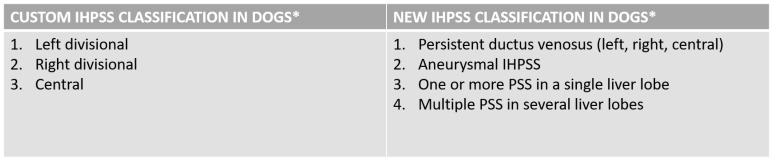
On the left, current classification of intrahepatic portosystemic shunt in dogs [54,55,56]. On the right, the new classification proposal. Four different phenotypes are possible. The persistent ductus venosus is the most commonly encountered IHPSS. It can be anatomically left, right or central located. Other complex IHPSS are increasingly recognized with CT and may involve one or more liver lobes, as already described in children [7,57].

**Figure 11:**
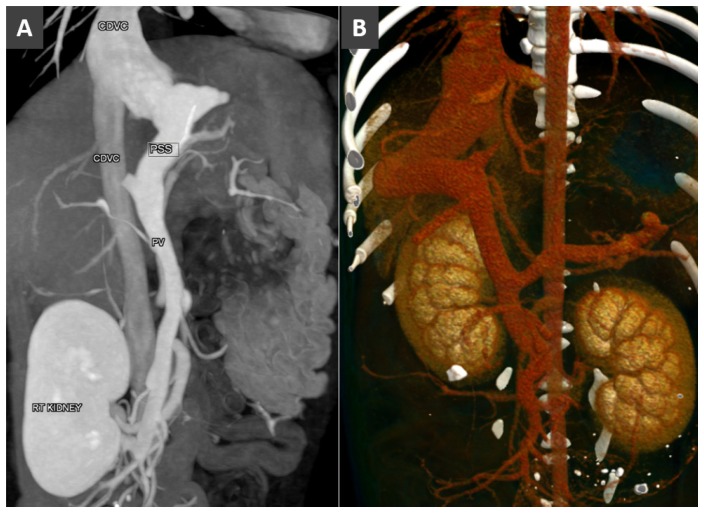
(**A**). Dorsal MIP in a dog with left-sided intrahepatic portosystemic shunt. (**B**). Volume rendered image (ventral view) in a dog with right-sided intrahepatic portosystemic shunt.

**Figure 12:**
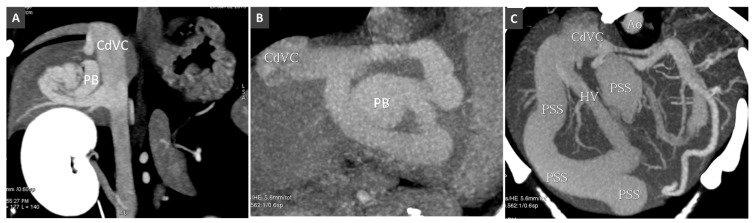
(**A**). Intrahepatic PSS in a dog involving a single hepatic lobe. PB, portal branch; CdVC, caudal vena cava. (**B**). Intrahepatic PSS through an aneurysmal dilatation of a portal branch (PB). (**C**). Multiple intrahepatic portosystemic connections involving several hepatic lobes.

CPSS refers to the presence of abnormal vascular connection between portal system and a systemic vein (of the caudal vena cava or azygous system), due to embryonic errors or foetal vascular persistence. Based on its anatomic location, CPSS are customarily divided into two main subtypes: intrahepatic and extrahepatic. *Intrahepatic portosystemic shunt (IHPSS)* mostly affects large-size dogs and generally represents a patent ductus venosus (foetal vascular persistence) [54,55,56]. Depending on the side of insertion into the hepatic caudal vena cava, three phenotypes are commonly described in the veterinary literature: right-sided, central and the most common left-sided (or -divisional) IHPSS (Figure 10). In humans, IHPSS are classified into four types based on the location of the shunt within the liver, number of connections and shunt characteristics [57]. With the widespread use of advanced imaging modalities, complex, multiple connections between portal branches and hepatic veins, affecting one or more liver lobes within the liver are increasingly described also in dogs (Figure 11 and Figure 12). Therefore, in the author’s opinion, the classification of IHPSS in dogs should be updated (Figure 10). Complex IHPSS are generally accompanied by critical clinical conditions and should be correctly identified before treating [45,58].

*Extrahepatic portosystemic shunts (EHPSSs)* are developmental anomalies resulting from anomalous connections between the vitelline veins, which form the portal system and the cardinal veins, forming the systemic veins. Although the genetic basis of CPSS in dogs has not been clearly established, many studies have demonstrated that this condition occurs more frequently in purebred dogs and that it is inherited in several small and toy breeds [7,59,60,61]

**Figure 13:**
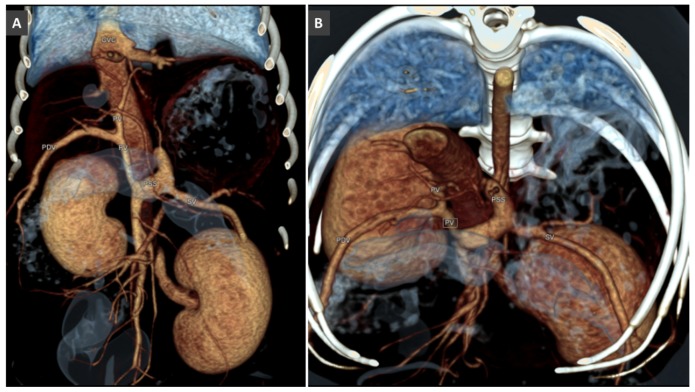
Extrahepatic PSS emanating from left gastric vein in a dog. (**A**). Volume rendered ventral view. (**B**). Frontal-oblique view, showing the site of insertion of the PSS in the pre-hepatic segment of the caudal vena cava.

**Figure 14:**
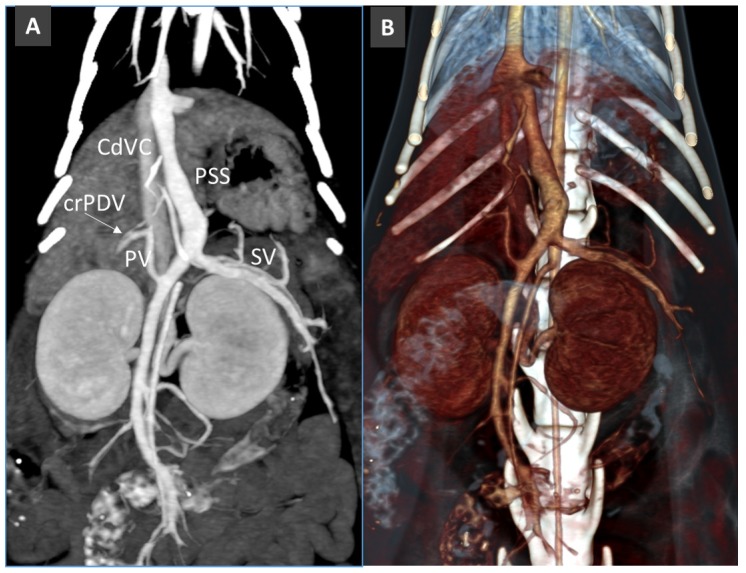
Congenital PSS from left gastric vein in a cat. Note the thin, hypoplastic portal vein (PV). (**A**). Maximum Intensity Projection. (**B**). Volume Rendering.

**Figure 15:**
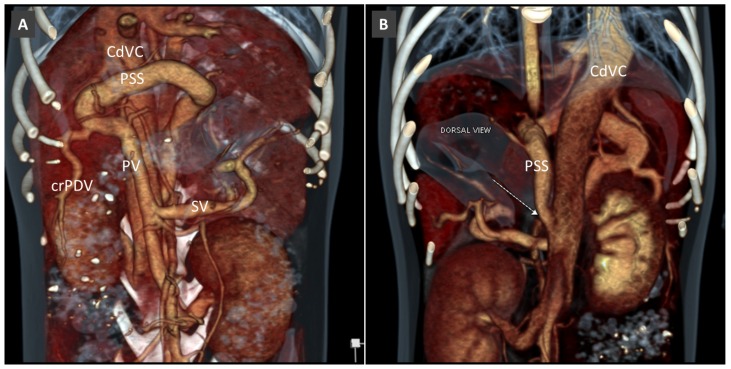
Extrahepatic congenital PSS from right gastric vein to the pre-hepatic segment of the caudal vena cava (CdVC). (**A**). Volume rendered image, ventral view (CrPDV, cranial pancreatic duodenal vein). (**B**). Dorsal view showing the site of the insertion of the PSS into the CdVC.

**Figure 16:**
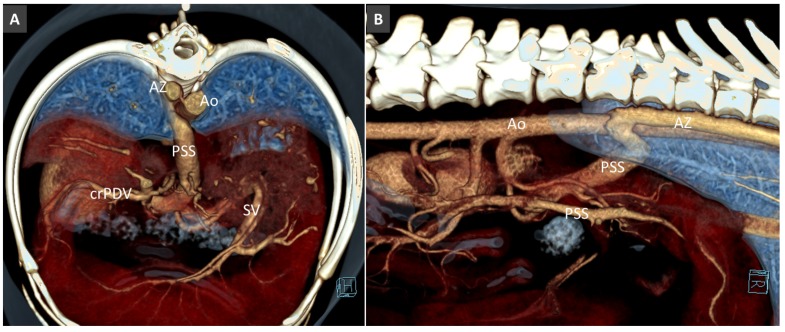
Congenital extrahepatic PSS in a dog, from left gastric vein to the right azygos vein (Az). (**A**). Volume rendered image, frontal view. (**B**). Right lateral view, showing the course and termination of the shunting vessel into the right azygous vein.

**Figure 17:**
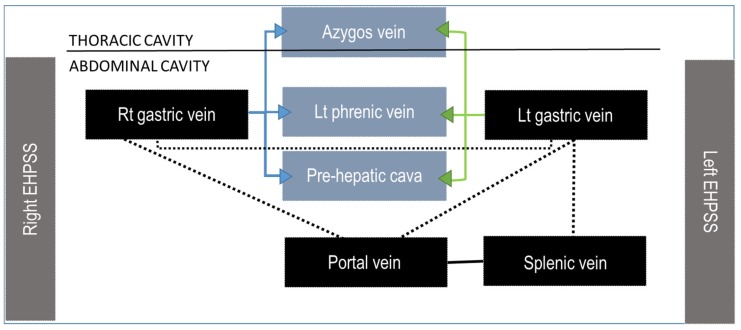
Most common patterns of side-to-side EHPSS reported in the veterinary literature. The black dotted lines represent the normal connections between the portal vein and its tributaries.

The initial description of Abernethy originally reported in people and the following functional classification of Morgan and Superina may be adopted also in veterinary patients (Figure 1) [30,31]. This classification system is based on whether the portal vein, often hypoplastic, is present and whether the liver is perfused with blood from the mesenteric venous system. These different types have different clinical impact, treatment options and outcome. In the type I or **end-to-side** EHPSS the connection is directly between the portal vein and a systemic vein due to CAPV and there is no discernible portal flow to the liver (Figure 3). The type II or **side-to-side** shunt, include a number of EHPSS between a portal tributary, most commonly emanating from left or right gastric veins and a systemic vein (cava or azygos vein) (Figure 13, Figure 14, Figure 15 and Figure 16). These connections most commonly are emanating from left or right gastric veins. (Figure 17) [61,62,63] The side-to-side is the most common type of CPSS described in veterinary patients [10,11,12,61,62,63]. Various repetitive phenotypes of side-to-side EHPSS, which reflect a common underlying embryological error, have been described in dog and cats.). Anomalous portosystemic connections can also involve the mesenteric tributaries of the portal vein system. Mesenterico-renal-caval shunt has been reported in dogs and left colic vein or cranial rectal vein to pelvic systemic vein communications (directly to the caudal vena cava or through common iliac vein or internal iliac vein) has been reported in both dogs and cats [63,64].

In side-to-side EHPSS, there is preservation of at least some hepatic portal flow [65,66]. Many of these patients have extremely hypoplastic portal vein cranial to the shunt that is sometimes difficult to visualize with conventional CT angiography. High quality, thin-slices MDCT images may help in detection of a thin portal vein and portal branches. These patients can be treated using partial shunt banding to allow gradual expansion of the intrahepatic portal system while avoiding irreversible portal hypertension. Splenosystemic shunts with the azygous vein, the left phrenic and left gonadal veins have been reported either among acquired or congenital patterns in dogs and cats and their definitive aetiology is still debated [10,11,12,47,67,68]. The presence of other direct or indirect signs of portal hypertension, such as ascites or varices, can help the interpretation of these vascular patterns. However, those cases without ascites or multiple portosystemic connections are more difficult to interpret [68]. 

The left or right gastric veins can emanate shunts joining the caudal vena cava, phrenic vein or the azygos vein directly or can connect with left spleno-gastric veins and then enter the systemic vasculature. Caudal tributaries contributing to the portal venous system, mesenteric and colic veins can connect directly to the caudal vena cava or enter the systemic circulation via renal vein or pelvic veins.

### 3.7. Acquired Portosystemic Shunts (APSSs) 

APSSs are characterized by hepatofugal pathways that can be caused by portal hypertension (increased resistance in the portal system) or increased resistance in the cranial vena cava system. In these cases, a combination of hemodynamic, anatomic and angiogenetic factors lead to neo-angiogenesis and the opening of pre-existing vascular connections between the portal and systemic circulations [69]. In normal mammals, no gross connection between these systems is present. However, at least three embryonic connections, with no or minimal perfusion, are present in normal animals that may enlarge in case of portal hypertension: the left colic–pudendal vein, left gastric–cardiac oesophageal branches and phrenic–portal vein connections [4,70].

**Figure 18:**
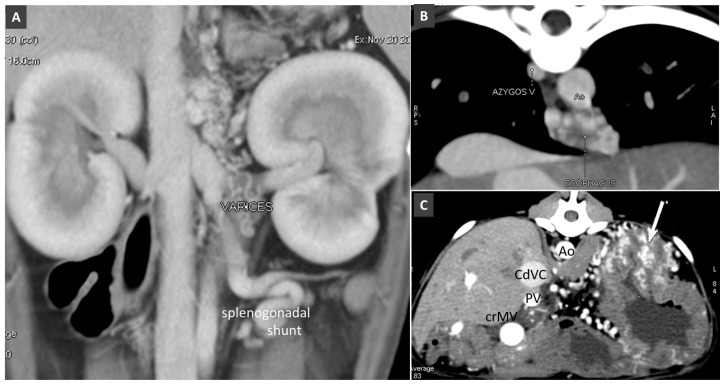
(**A**). Dorsal thin-MIP showing small varices in left retroperitoneal region (gastrophrenic varices) and splenogonadal shunt in a dog with portal hypertension. (**B**). Transverse view at the diaphragmatic level in a dog with portal hypertension and oesophageal varices. (**C**). Transverse view of the cranial abdomen in a dog with portal hypertension due to HAVM. Arrow indicates gastric submucosal varices. Ao, aorta; CdVC, caudal vena cava; PV, portal vein; crMV, cranial mesenteric vein.

**Figure 19:**
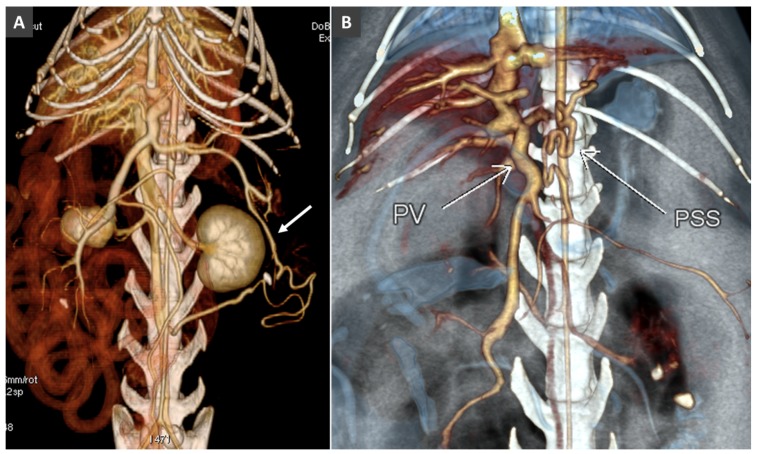
(**A**). Splenogonadal shunt in a cat with portal hypertension. (**B**). Splenophrenic shunt in another cat with portal hypertension.

Several patterns of APSS have been reported in dogs and cats [10,70,71] They can be divided grossly into large shunts (e.g., left splenogonadal or splenophrenic shunt) and small shunts (e.g., oesophageal, gastrophrenic or colic varices). Large and small APSS often coexist in the same patient (Figure 18 and Figure 19). Varices may be subdivided according to their anatomic location and pathways into left gastric vein, gastrophrenic, omental, gallbladder, abdominal wall, duodenal and colic varices [10,72]

Importantly, anomalous vascular connections between systemic and portal systems can also occur in case of chronic obstruction of the caudal vena cava (cavo-portal collaterals) allowing blood to return to the right atrium [73]. Special care should be taken to not misinterpret these vascular anomalies.

### 3.8. Porto-Portal Collaterals or Cavernous Transformation of the Portal Vein

The gradual thrombosis or extrinsic compression of the portal vein stem may lead to obstruction of the portal vein that will result partially absent on MDCT images. Portal vein obstruction causes portal hypertension and can be accompanied by portal cavernous transformation and acquired portosystemic shunts (APSSs) [10,47].

**Figure 20:**
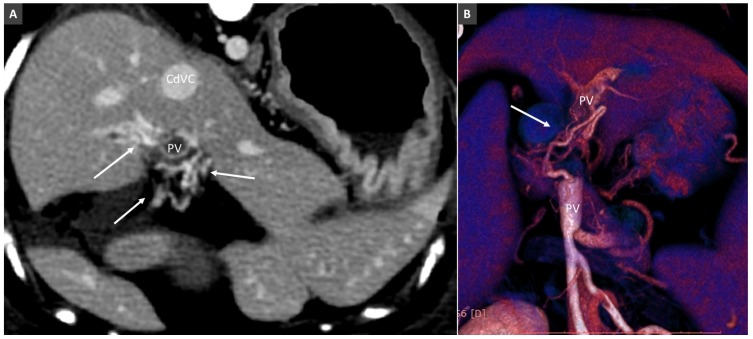
Porto-portal collaterals (cavernous transformation of PV) in a dog due to PV thrombosis. (**A**). Transverse view at the porta hepatis, showing multiple tortuous vessels surrounding the thrombosed PV (arrows). (**B**). Volume rendered image (ventral view) in another dog with extrahepatic PV thrombosis. Arrow indicates a porto-portal collateral bypassing the site of obstruction.

Cavernous transformation of the portal vein (CTPV) refers to the radiological appearance of porto-portal collaterals around a thrombosed portal vein [47]. Two main types of porto-portal collaterals are described in dogs and cats: short tortuous collaterals developing around/inside the thrombus and long collaterals, forming a network to overcome the obstructed portal vein (Figure 20). These collaterals course the hepatoduodenal ligament, around the gallbladder and common biliary and cystic ducts and terminating in portal branches within the liver. When complete portal vein obstruction persists for some time, portal hypertension can ensue and hepatofugal portosystemic collateral pathways will develop. Portal obstruction and indirect signs of portal hypertension must be distinguished from the congenital absence of the portal vein eventually associated with congenital portosystemic shunts (CPSS) [33]. The closure of acquired portal collaterals in patients with portal hypertension may be fatal.

## 4. Conclusions

Portal vein system anomalies are numerous and often related to each other. Correct identification of these vascular anomalies is important because they have different clinical and prognostic significance and is essential for selecting appropriate treatment options.
